# Microbiological Evaluation of Two Mexican Artisanal Cheeses: Analysis of Foodborne Pathogenic Bacteria in Cotija Cheese and Bola de Ocosingo Cheese by qPCR

**DOI:** 10.3390/foods13172824

**Published:** 2024-09-05

**Authors:** Cindy Adriana Estrada-Hernández, María Belén Becerra-Cedillo, Irma Angélica Hernández Velázquez, Hermann E. Mejía-Buenfil, Tania Olivera-Martínez, I. Berenice Salto-González, Frida Torres-López, Maricarmen Quirasco

**Affiliations:** Food and Biotechnology Department, School of Chemistry, National Autonomous University of Mexico, Ciudad Universitaria, Mexico City 04510, Mexico; cindyaeh@comunidad.unam.mx (C.A.E.-H.);

**Keywords:** Cotija cheese, Bola de Ocosingo cheese, qPCR, foodborne pathogenic bacteria, ripened cheese, food safety

## Abstract

Cotija and Bola de Ocosingo are artisanal ripened cheeses produced in Mexico. Both are made with raw bovine milk from free-grazing cows and with no starter cultures. Unlike culture-based techniques, molecular methods for pathogen detection in food allow a shorter turnaround time, higher detection specificity, and represent a lower microbiological risk for the analyst. In the present investigation, we analyzed 111 cheese samples (95 Cotija and 16 Bola de Ocosingo) by qPCR (TaqMan^®^) after an enrichment-culture step specific to each foodborne bacterium. The results showed that 100% of the samples were free of DNA from *Listeria monocytogenes*, *Brucella* spp., *Escherichia coli* enterotoxigenic (ETEC), and O157:H7; 9% amplified *Salmonella* spp. DNA; and 11.7%, *Staphylococcus aureus* DNA. However, the threshold cycle (Ct) values of the amplified targets ranged between 23 and 30, indicating DNA from non-viable microorganisms. Plate counts supported this assumption. In conclusion, 100% of the cheeses analyzed were safe to consume, and the enrichment step before DNA extraction proved essential to discern between viable and non-viable microorganisms. Hygienic milking, milk handling, cheese manufacturing, and ripening are crucial to achieve an adequate microbiological quality of cheeses made with raw milk.

## 1. Introduction

In Mexico, the Institute of Industrial Property (*Instituto Mexicano de la Propiedad Industrial*; IMPI, in Spanish) confers the label known as *Marca Colectiva* (Collective Brand) to products made by members by any established association based on the geographic origin, manufacturing process, raw materials, and other distinctive characteristics. Currently, four artisanal cheeses have the *Marca Colectiva* label granted by the IMPI, namely, Cotija, Poro de Balancán, Cuadro, and Bola de Ocosingo [1].

Recently, consumer demand for minimally processed foods has increased. The raw materials of Cotija and Bola de Ocosingo cheeses are only milk, salt, and rennet, so they could belong to this group [2]. Furthermore, since farms that produce these cheeses are in geographic areas free of pesticides, they could fulfill standards for future organic certification. Therefore, these products are an attractive alternative in these markets.

**Figure 1:**
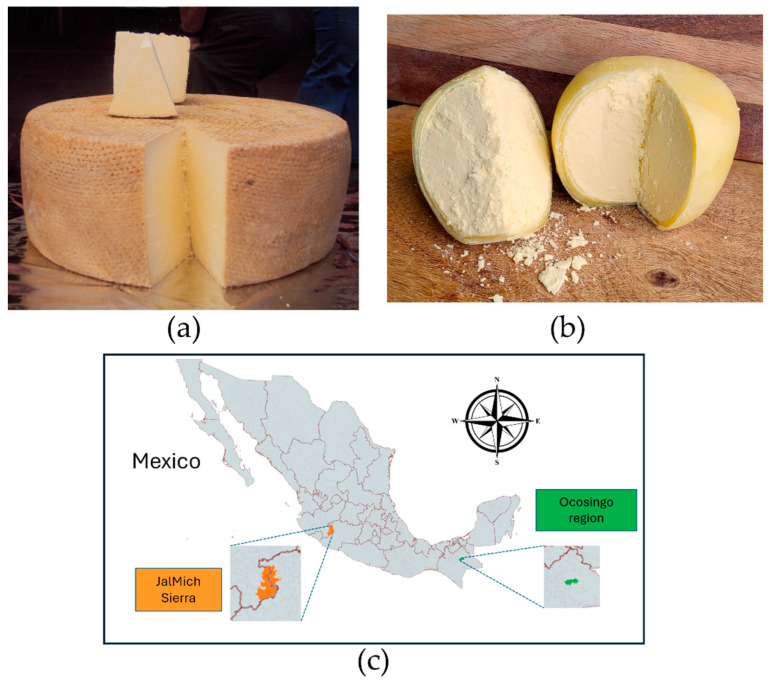
(**a**) Cotija cheese ripened for one year (photograph by M. Quirasco). (**b**) Bola de Ocosingo cheese (photograph by C. Estrada). (**c**) Location of artisanal cheese production areas in Mexico.

Cotija cheese is a dairy product manufactured during the rainy season (July–October) in the mountains between the states of Jalisco and Michoacán (JalMich Sierra, 700–1700 m above sea level) (Figure 1c). It is manufactured traditionally, which dates back to the arrival of the Spaniards to Mexico. It is a pressed, hard, and uncooked-paste cheese. It has an intense and penetrating aroma, crumbly texture, high-fat content, and strong and acidic flavor. It is a large-format cheese that may weigh up to 30 kg per piece (Figure 1a). Its production process involves raw milk curding, curd cutting, dry salting, kneading, pressing, and a ripening stage from 3 months to one year at room conditions (23–25 °C and 60–95% relative humidity, RH) [3,4]. Due to its high cultural and commercial value in the Cotija region and abroad, efforts are underway to obtain the Protected Designation of Origin [5].

Bola cheese is produced throughout the year in the municipality of Ocosingo (880 m above sea level), in the southern state of Chiapas, close to the Montes Azules rainforest and the border with Guatemala (Figure 1c). It is a double-cream cheese made from raw cow milk, with a semi-hard, creamy, and crumbly texture (hereafter referred to as core). It has a rind of a double layer of string cheese made with skim milk. This double layer stiffens when dried, thus serving as a sort of package that extends the shelf life of the core. Bola cheese sells in 400 g and 1 kg pieces (Figure 1b). The core cheese is left hanging inside a clean cloth for 21 days to ripen at room conditions. In the Ocosingo area, the mean temperature and RH are 13 °C to 27 °C and 70%, respectively, in the dry season (March–April) and 15 °C to 25 °C and 88% RH in the rainy season (September–October). After ripening, the farmhouse producer covers the core with two layers of string cheese, shaping it as a ball. Afterwards, it is stored at 4 °C until consumption [3,4].

Tracking pathogenic bacteria that could endanger cheese safety is important because both cheeses are produced with raw cow milk. In terms of regulations, the Mexican Official Standard (Milk and Dairy Products—Sanitary Specifications and Test Methods) establishes that pasteurized milk shall be used to manufacture dairy products [6]; however, some exceptions are considered in a recent, still unpublished review, such as ripened cheeses. Additionally, the Mexican Standard NMX-F-735-COFOCALEC-2018 (Regional Dairy Product—Ripened Artisanal Cotija Cheese—Designation, Specifications, and Test Methods) considers the use of raw milk in the production of Cotija cheese, recognizing that the native microbiota contributes to the final organoleptic characteristics of cheese and acknowledging its cultural and artisanal attributes [7].

While cheese-making with pasteurized milk minimizes the risk of foodborne diseases, the microbiota in raw milk is essential to provide flavor and aroma notes in ripened cheeses [8,9,10]. Furthermore, cheese made from unpasteurized milk does not necessarily pose a risk for human consumption, as this will depend on five relevant factors: the quality of the raw material, the hygiene practices in place during manufacturing, the ripening process, the storage conditions of the finished product, and hygiene at the point of sale [8,11,12].

Despite the efforts of producer associations to implement processes following good manufacturing practices, assessing the microbiological quality of such products will always be critical, as it provides information on the safety of artisanal cheeses made from unpasteurized milk. This information is also of interest to consumers and governmental health authorities. The Mexican Official Standards that establish microbiological guidelines for accepting or rejecting dairy products establish culture-based and biochemical assays. Currently, in the European Union, the use of microbial detection kits based on quantitative PCR (qPCR) multiplexing, loop-mediated isothermal amplification (LAMP), biosensor systems, or microarrays is more common, as molecular techniques provide faster results and are accurate, specific, robust, and sensitive enough [13,14,15].

The real-time quantitative PCR method, also known as qPCR, is a technique with high sensitivity whose specificity is set by the design of the oligonucleotide primers and probes used in the reaction. Furthermore, its specificity increases when the TaqMan^®^ fluorogenic probe hydrolysis chemistry is used instead of the SYBR green intercalating fluorophore chemistry [16,17]. Unlike traditional microbiological techniques, molecular approaches such as qPCR offer shorter turnaround times and avoid microorganism handling in plate streaking and confirmation by biochemical or serological tests, thus significantly reducing the risk of infections in diagnostic or research laboratory personnel [18].

This work aimed to analyze the microbiological quality of 95 samples of Cotija cheese and 16 samples of Bola de Ocosingo cheese through the targeted detection of five pathogenic microorganisms of interest in dairy foods: *Salmonella enterica*, *Escherichia coli* enterotoxigenic (ETEC), *E. coli* O157:H7, *Listeria monocytogenes*, *Staphylococcus aureus*, and *Brucella abortus* through real-time PCR.

## 2. Materials and Methods

### 2.1. Biological Materials

The bacterial strains used in this investigation were *Salmonella enterica* Typhimurium (ATCC 14028), *Staphylococcus aureus* (ATCC 6538), *Escherichia coli* DH5α (Invitrogen, Carlsbad, CA, USA), *Limosilactobacillus fermentum* (isolated from Bola de Ocosingo cheese and identified in our laboratory by 16S sequencing), *Brucella abortus* (a pathogenic strain kindly donated by Dr. Beatriz Arellano, School of Veterinary Medicine and Zootechnics, Microbiology and Immunology Department, National Autonomous University of Mexico)*, Listeria monocytogenes* (CFQ-B-103), *E. coli* O157:H7 (CFQ-B-260), *E. coli* ETEC (CFQ-B-296), *Enterococcus faecalis* (CFQ-B-254), and *E. faecium* (CFQ-B-255). The CFQ code corresponds to the Culture Collection of the School of Chemistry (CFQ) at the National Autonomous University of Mexico (WDCM No. 100).

### 2.2. Cheese Samples

Ninety-five 3 kg Cotija cheese samples were purchased from different farms in the municipalities mentioned in the Mexican Official Standard (NMX-F-735-COFOCALEC, 2018). These samples were stored in a tightly sealed bag and transported in a cooler to the laboratory; once there, they were kept at −70 °C until analysis. After defrosting at 4 °C for 18 h, the rind (inedible part) was removed with sterile knives in a laminar flow cabinet, and the cheese was ground with a food processor. Finally, the homogenized cheese samples were stored in sterile resealable plastic bags at −20 °C until analysis.

Sixteen Bola de Ocosingo cheeses were purchased as individual pieces, with the rind intact, from eight different producers in the city of Ocosingo, Chiapas. These cheeses were transported in coolers to the laboratory, where they were kept frozen at −20 °C until analysis. A portion of the external rind was removed with a sterile knife to collect a homogeneous sample of the core cheese in a laminar flow cabinet.

Spatulas, knives, processor blades, and other instruments were washed with soap and water, cleaned with a 10% (*v*/*v*) sodium hypochlorite solution and 75% ethanol, and autoclaved to avoid cross-contamination between samples. The food processor vessel could not be heat-sterilized, so after washing with soap and cleaning with sodium hypochlorite and ethanol, it was left in the UV cabinet (λ = 254 nm) for 5 min.

### 2.3. Culture of Reference Strains and Bacterial Enrichment in Cheese

The pathogenic strains used as reference were cultivated under the conditions mentioned in Table 1. *E. faecalis*, *E. faecium,* and *L. fermentum* were cultivated in Man, Rogosa, and Sharpe (MRS) medium (OXOID, Basingstoke, UK) for 16 h at 250 rpm and 37 °C. *E. coli* DH5α was cultured in Brain Heart Infusion (BHI) broth (DIFCO, Detroit, MI, USA) overnight at 37 °C in an incubator (static).

Twenty-five grams of cheese was homogenized in a Stomacher 400 circulator (Seward Laboratory, London, UK) at high speed for 5 min with 225 mL of diluent and 1 mL of Neutrase solution (Novozymes Latin America, Paraná, Brazil). For *E. coli*, *Salmonella* spp., and *L. monocytogenes*, the diluent was 2% sodium citrate, pH 8.0. For *S. aureus* and *Brucella* spp., buffered peptone water, pH 7.0, was used. Subsequently, the mixture was incubated for 1 h at 45 °C to allow Neutrase proteolysis. Forty milliliters of the mixture was collected and centrifuged at 1500× *g* for 5 min at 4 °C; this procedure was performed in duplicate for each sample. Avoiding the upper-fat layer and solids at the bottom, 2.5 mL of the intermediate liquid phase was extracted and transferred to 25 mL of the enrichment broth corresponding to each pathogenic microorganism in order to increase the concentration of the target pathogen in the sample. The mixtures were incubated under the conditions indicated in Table 1.

### 2.4. DNA Extraction

After incubation, cultures were heat-inactivated for 20 min at 80 °C and centrifuged at 10,000× *g* for 10 min at 4 °C. The pellet was washed twice with 500 µL sterile saline, pH 7, and centrifuged at 10,000× *g* for 10 min at 4 °C. Then, it was stored at −20 °C until nucleic acid extraction. DNA extraction was performed with the Fast ID Genomic DNA extraction kit (Genetic ID NA, Inc., Fairfield, IA, USA) or the DNeasy Mericon Food Kit (Qiagen, Germantown, MD, USA); in both systems, cell disruption is achieved using detergents and lytic enzymes. DNA was purified with chloroform before using silica columns and was eluted with 50 µL H_2_O for molecular biology (MoBio, Carlsbad, CA, USA). The extracted DNA was stored at −20 °C until quantification. DNA analysis was performed on an EPOCH plate reader (BioTek, Winooski, VT, USA).

### 2.5. qPCR Reaction

The gene sequences for toxin and antigenic proteins specific to each pathogenic bacterium were considered targets in the design of the primers and probes used in qPCR (Table 2).

The qPCR procedures follow the general requirements of ISO 22174 and the protocols of the World Organization for Animal Health (WOAH) [25,26]. For Cotija cheese analyses, the enzyme used for the reaction mixture was Universal Master Mix TaqMan PCR with ROX (Applied Biosystems, ABI, Foster City, CA, USA) at a 1X final concentration. For *Salmonella* spp., *S. aureus,* and *L. monocytogenes*, primers and probes were purchased in a 20X stock mix (Gene Expression Assay, ABI, Foster City, CA, USA), diluted to a 1X final concentration. For *Brucella* spp., *E. coli* ETEC, and *E. coli* O157:H7, the primers and probe were used at 300 nM and 180 nM, respectively (ABI, Foster City, CA, USA). For the Bola de Ocosingo cheese analyses, the enzyme used in the reaction mixture was RealQ Plus 2X Master Mix for the probe with ROX (Ampliqon, Odense, Denmark), diluted to a 1X final concentration. The primers and probe were used at 300 nM and 180 nM, respectively (Integrated DNA Technologies, IDT, Coralville, IA, USA). For the analysis of both cheeses, qPCR was carried out in a final reaction mix volume of 20 µL, and the amount of template DNA analyzed was 100 ng per reaction. The programming on the ABI 7500 (ABI, Foster City, CA, USA) platform was as follows: one cycle at 50 °C/2 min, 1 cycle at 95 °C/10 min, and 40 cycles (95 °C/15 s, 60 °C/1 min). Fluorescence data were collected at the end of the extension step and analyzed with 7500 Real-Time PCR Software v2.3 and QuantStudio Real-Time PCR Software v1.3 (ABI, Foster City, CA, USA). Non-template controls (NTCs) (H_2_O for molecular biology, MoBio, Carlsbad, CA, USA) and positive amplification controls (DNA from the respective pathogenic bacterium as template) were added to each qPCR plate in duplicate. The threshold line was set in the exponential phase of the amplification step where the data of replicates were reproducible [27].

A summary of the cheese analysis pipeline is as follows: The bacterial population was enriched in differential cultures for each cheese sample in duplicate. Then, DNA was extracted from the cell pellet. Then, qPCR was performed on each extracted DNA in duplicate. Finally, four qPCR results were obtained from each cheese sample.

### 2.6. Assessment of Primer–Probe Specificity and Colony Forming Unit (CFU) Limit of Detection

To evaluate cross-reactivity, i.e., to assess false positives, qPCR was performed using DNA from a microorganism other than the target gene of the primers and probe set. The reactions were performed in quadruples.

To evaluate the CFU limit of detection, a colony of *S. enterica* in BHI (Difco, Detroit, MI, USA) (approximately 3 mm in diameter) was sampled and vortexed in 10 mL of sterile saline for 15 s. From this solution (10^−1^ dilution), ten-fold serial dilutions up to 10^−10^ were prepared. One set of dilutions was plate-counted in BHI (37 °C, 24 h). Another set was heat-inactivated, and then 1 mL of each dilution was inoculated into 25 mL of buffered peptone water, adding 2.5 mL of the intermediate phase after the centrifugation of the homogenized cheese on peptone water (Section 2.3). Each mixture was incubated under the conditions for *S. enterica* (Table 1). Then, cultures were heat-inactivated, and DNA extraction and qPCR were performed in duplicate following the same procedures described in Section 2.4 and Section 2.5, respectively. For this experiment, the cheese used for artificial contamination was the one in which no qPCR amplification was observed in a previous analysis.

### 2.7. qPCR of Spiked Microorganisms in Cheese

In separate experiments, a colony of each pathogenic bacteria was vortexed in 10 mL of sterile saline for 15 s, and then serial ten-fold dilutions up to 10^−8^ were prepared. One set of dilutions was plate-counted in BHI (37 °C, 24 h). From the homogenized 10^−8^ dilution, 1 mL was collected and inoculated into 25 mL of culture medium corresponding to each pathogen (Table 1), adding 2.5 mL of the intermediate phase after the centrifugation of the homogenized cheese on peptone water as described in Section 2.6. Cultures were incubated under the respective conditions for each pathogenic bacterium (Table 1). Afterward, each culture was heat-inactivated, and DNA extraction and qPCR were performed in duplicate following the procedure described above. Another set of experiments was performed following the same methodology but spiking a heat-inactivated inoculum.

### 2.8. Endpoint PCR

To evaluate the presence of inhibitors and DNA integrity, endpoint PCR reactions were performed to amplify the bacterial 16S ribosomal gene (rDNA) V3 region. The reaction mixture consisted of Taq DNA polymerase (Ampliqon, Odense, Denmark), 0.5 U per reaction, Taq buffer Mg_2_SO_4_ 1X, the forward primer 338f (5′-ACT CCT ACG GGA GGC AGC AG-3′), and the reverse primer 518r (5′-ATT ACC GCG GCT GCT GCT GCT GG-3′) [28]; both primers were at 0.5 µM and 100 ng of DNA as a template. The final mixture was brought to 50 µL with PCR-grade water, and the reaction conditions on the MaxyGene thermal cycler (Axygen Scientific, Union City, CA, USA) were as follows: 1 cycle at 94 °C for 5 min, 20 cycles (94 °C, 1 min/65 °C, 1 min/72 °C, 3 min), 10 cycles (94 °C, 1 min/55 °C, 1 min/72 °C, 3 min), and, finally, 1 cycle at 72 °C for 10 min. PCR products were visualized on 2% agarose gels stained with ethidium bromide at 0.5 µg/mL using the Mass Ruler Low Range DNA Ladder (Fermentas International, Vilnius, Lithuania) or the GeneRuler 1 kb DNA ladder (Thermo Fisher Scientific, San Jose, CA, USA) as references. Non-template controls (NTCs) (H_2_O for molecular biology, MoBio, Carlsbad, CA, USA) and positive amplification controls (DNA from different bacterium as template) were included in the analysis.

### 2.9. Viable S. aureus Assessment

A culture-dependent analysis of *S. aureus* was performed on cheeses that tested positive in qPCR. The enumeration was performed on Baird Parker ready-to-use plates (Becton Dickinson and Co., Franklin Lakes, NJ, USA) (37 °C, 48 h) [29] in duplicate. Typical *S. aureus* colonies are black, circular, shiny, convex, smooth, and 1–2 mm in diameter and display an opaque zone and a clear halo around them.

## 3. Results and Discussion

There is scarce information on the safety of ripened cheeses made with raw milk in Mexico and Latin America. In this work, the microbiological safety of two traditional Mexican products is evaluated to contribute to a better understanding of the relevance of the ripening process of cheeses made with raw milk and to provide information to consumers on the safety of these kinds of products [30,31]. These concerns motivated us to test for the presence of the most relevant foodborne pathogenic bacteria in dairy products, namely, *S. enterica*, *S. aureus*, *L. monocytogenes*, *B. abortus*, *E. coli* ETEC, and *E. coli* O157:H7 in two artisanal ripened cheeses made in Mexico: Cotija and Bola de Ocosingo. On the other hand, the implementation of culture-independent methods for the microbiological analysis of food in our region is limited; therefore, we propose a molecular assay that is sensitive and less laborious, with a high-throughput capacity and shorter turnaround time, namely, qPCR.

The *Brucella* primer–probe set was designed taking *per* as the target gene, which is common in several species of that genus, including *B. abortus*, which infects bovine cattle. The other primer–probe sets have a species-specific target gene, and in the case of *E. coli*, they differentiate between ETEC and O157:H7 types. Consequently, the first step was to verify the specificity of the primer–probe sets using DNA from pure cultures of the collection strains. In the experimental protocol, we also included testing for cross-reactivity with DNA from microorganisms isolated from each cheese. This is the case for *E. faecium*, *E. faecalis*, and *L. fermentum*, which are part of the cheese microbiota [8,32]. We also tested the DNA of a non-pathogenic strain of *E. coli*, i.e., DH5α.

### 3.1. Primer–Probe Set Cross-Reactivity

First, the in silico specificity of the primer–probe sets was evaluated against the database available in GenBank using BLAST (Basic Local Alignment Search Tool) [33]. Subsequently, the primer–probe sets’ cross-reactivity was experimentally evaluated; the results are shown in Table 3. The amplification of DNA from the pure cultures of strains generated amplification curves with threshold cycle (Ct) values between 11 and 14. When DNA from microorganisms other than the target was used as a template, no amplification or curves with Ct values higher than 30 were obtained, which can be attributed to the formation of primer dimers or other non-specific products [34].

The specificity test results were useful in confirming that the primers and probes designed for each bacterium only produce amplification signals when analyzing DNA from that specific pathogen and give null signals or high Ct values when testing DNA from other microorganisms, including those that could be found naturally in cheese, such as the genus *Enterococcus* and *Lactobacillus* [8,32,35]. Therefore, an optimal design of oligonucleotide primers and fluorogenic probes is crucial to avoid non-specific amplifications (false positives caused by PCR artifacts) and obtain reliable results.

### 3.2. Limit of CFU Detection Assessment

**Figure 2:**
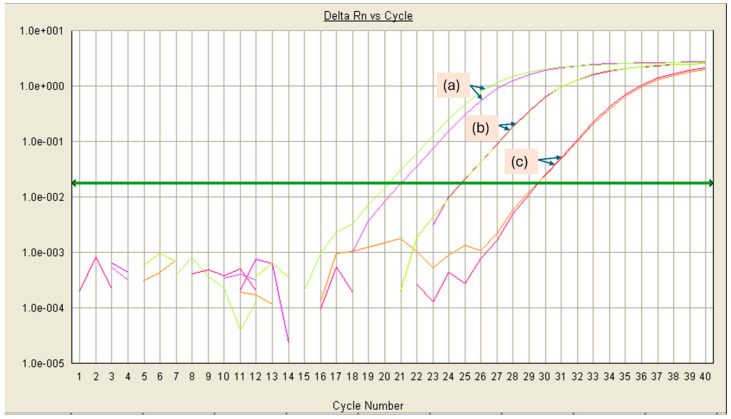
qPCR amplification plots of ten-fold serial dilutions of non-viable *S. enterica* spiked in Cotija cheese. (a) 10^−6^, (b) 10^−7^, and (c) 10^−8^ dilutions, respectively. Reactions were performed in duplicate.

Figure 2 shows the amplification plots of DNA obtained after inoculating cheese with ten-fold dilutions of non-viable *S. enterica*. It is observed that the difference in Ct values between each curve corresponds to approximately four cycles; at dilutions higher than 10^−8^, the plots obtained were no longer reproducible or did not show amplification at all. The amplification curve of the *S. enterica* 10^−7^ dilution with a Ct value of 24.73 ± 0.04 corresponds to a plate count of 9 CFU/g cheese. Due to the exponential nature of the PCR, the following curve (Figure 2c, Ct = 29.48 ± 0.05) would correspond roughly to 1 CFU/g cheese. Considering that the target genes for amplification are in a single copy in the genomes of the microorganisms of interest, a Ct < 30 was interpreted as a positive result, comparable to the one used by Mendonça et al. [36].

The exponential amplification principle of PCR can explain the difference of approximately four cycles between each 10-fold dilution [17,37]. Theoretically, a ΔCt of 3.32 cycles would be expected for a ten-fold template dilution. However, we did not use pure DNA; instead, we inoculated thermally inactivated microorganisms in the cheese matrix, which went through the analysis pipeline (incubation, the recovery of the cell pellet, and DNA extraction). Kadiroğlu et al. reported a similar effect on the detection of *S. aureus* in artificially contaminated white cheese [38].

### 3.3. Effect of the Cheese Matrix on Pathogen Detection

The decimal dilutions 10^−6^ to 10^−10^ of the reference strains were plate-counted, and, in agreement with our previous result, it was determined that inoculating 1 mL of the 10^−8^ dilution was equivalent to having approximately 4 CFU/g cheese. Therefore, that would be the minimum amount of the pathogen that could be intentionally spiked in cheese to assess its presence with the qPCR protocol. After artificially contaminating each pathogenic bacteria, the samples were processed following our established pipeline, so this procedure would also evaluate the suitability of the culture-enrichment protocol and the presence of PCR inhibitors in the cheese matrix. The results are shown in Table 4. A control was also analyzed with DNA from the uninoculated cheese, in which no amplification was obtained with any primer–probe set.

**Figure 3:**
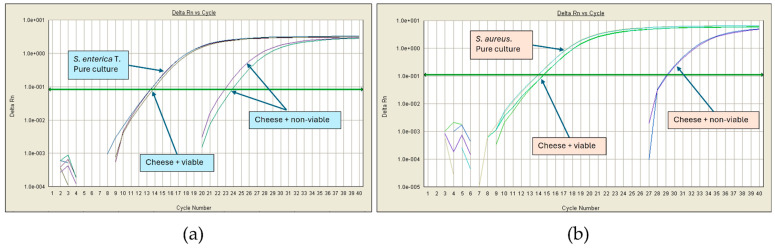
qPCR amplification plots using viable and non-viable microorganisms spiked into cheese. (**a**) Template DNA from *S. enterica*. (**b**) Template DNA from *S. aureus*.

An additional experiment was performed by spiking heat-inactivated microorganisms. After following the pipeline analysis, a Ct value between 23 and 29 was observed for all pathogens. Figure 3 shows the amplification plots of artificial cheese contamination with viable and non-viable *S. enterica* and *S. aureus*.

These results show that the media and enrichment conditions are appropriate; therefore, even if there were 4 CFU/g cheese, they could grow the equivalent of four logarithmic units, so the Ct obtained would be very similar to that of the pure strains. On the other hand, the system can detect DNA from non-viable cells, which, coming from bacteria that cannot grow, generate an amplification curve with a delayed Ct value > 23.

There are reports where the qPCR technique without the enrichment step is used to detect various pathogens in water and food; however, one disadvantage of such methods is that viable and non-viable bacteria cannot be differentiated [18,39]. Another disadvantage is that if the pathogen is in low quantities, it would return a false-negative result. Therefore, to increase the sensitivity of the qPCR detection method, an option would be to enrich the sample with the microorganism of interest either by the pre-enrichment of the sample in culture media or by other means prior to the qPCR; e.g., Garrido-Maestu et al. used magnetic nanoparticles functionalized with antibodies specific for some protein of the pathogen of interest in a step before qPCR [37,40]. However, considering a routine high-sensitivity application to qualitatively determine the presence of viable pathogenic bacteria, performing the enrichment step in culture media does not involve considerable time and costly resources. Increasing sensitivity (or LOD) is particularly important for *L. monocytogenes* and *S. enterica*, which cannot be present in food [41,42].

### 3.4. Cheese Analysis: DNA Quality Assessment

**Figure 4:**
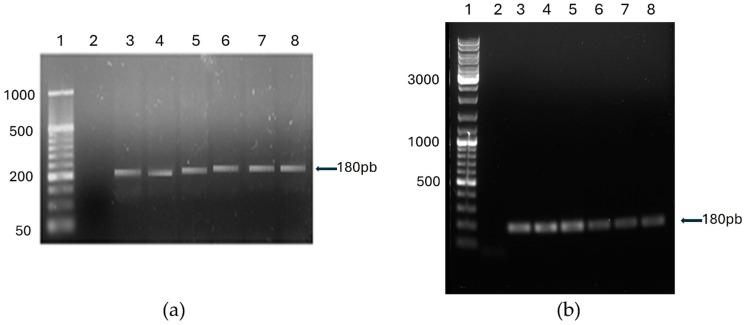
Amplification of the bacterial 16S rDNA V3 region. Bacterial DNA extracted from (**a**) Cotija cheese. Lanes: 1, MassRulerTM Low Range DNA Ladder (Fermentas); 2, NTC; 3–7, cheese samples 91–95; 8, positive control (DNA from *E. faecium*). (**b**) Bola de Ocosingo cheese. Lanes: 1, GeneRuler DNA Ladder Mix (Thermo Fisher Scientific); 2, NTC; 3–5, cheese samples 33, 44, and 90; 6–8, positive controls (DNA from *S. aureus*, *E. coli* ETEC, and *E. coli* O157:H7, respectively). Two percent agarose gel.

The molecular analysis of the 111 cheese samples begins with evaluating the amplification quality of the DNA extracted from the pelleted cells obtained after the enrichment step. DNA was obtained at a concentration between 10 and 200 ng/µL and had an acceptable purity according to the A_260_/A_280_ ratio (values of 1.8–2.2) [43]; however, some samples had values below 1.8. Therefore, to verify its quality as a template in a PCR reaction, the bacterial 16S rDNA V3 region was amplified. The PCR was also performed with other randomly selected good-quality DNA extracts as templates. An amplicon of the expected size, approximately 180 bp [44], was obtained in all analyzed preparations. DNA from pure cultures of reference strains was used as a template in positive-control reactions (Figure 4).

The amplicon analysis shows that after the bacterial enrichment step in the cheese samples, the DNA extracted from the cell pellet is of amplifiable quality, and there are no inhibitors that could prevent the action of the DNA polymerase. This means that a negative qPCR amplification result would not be due to the presence of any inhibitor in the reaction mixture or to the DNA being in small fragments that would prevent obtaining amplicons of approximately 200 bp, as in the case of the qPCR amplicons predicted for our primer–probe sets.

A critical aspect of food analysis by molecular techniques is obtaining DNA of the appropriate quality. The evaluation of the DNA extraction and purification method is of great importance for PCR applications in the food industry as the matrices frequently contain components that can inhibit the action of DNA polymerase. The former is of the utmost relevance in our protocol, considering that the starting matrix is cheese with high fat, protein, and Ca^2+^ contents, as these food components can inhibit the PCR reaction [38,45].

Note that a control without template DNA (NTC) was included in all PCR procedures. The absence of amplification in the NTCs indicated that the reagents were contaminant-free, so there was no chance of false positives. Likewise, samples and reagents were checked for proper handling, e.g., during pipetting, which showed no cross-contamination.

### 3.5. Cheese Analysis: Pathogen Detection

Table 5 summarizes the results obtained for both cheeses. For *L. monocytogenes*, *Brucella* spp., and *E. coli* (ETEC or O157:H7), there was no amplification or, if any, the Ct obtained was >30. Therefore, none of the cheese samples has DNA from any of those microorganisms.

**Figure 5:**
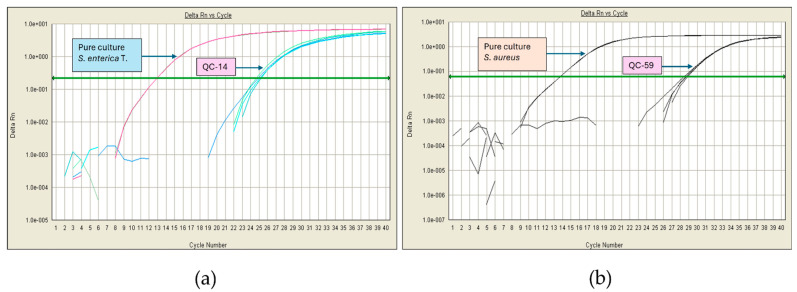
Examples of positive DNA detection of pathogenic bacteria in Cotija cheese by qPCR. (**a**) Amplification plots of *S. enterica*. (**b**) Amplification plots of *S. aureus*.

Regarding the screening of *S. enterica* and *S. aureus* in Cotija cheese, we observed DNA amplification in all four replicates in 10.5% and 13.7% of the samples, respectively. In the Bola de Ocosingo cheese, there was also no DNA amplification from these bacteria. Table 6 shows the qPCR results for samples that tested positive for *Salmonella* and *S. aureus* DNA, and Figure 5 shows the amplification plots of some positive results.

**Figure 6:**
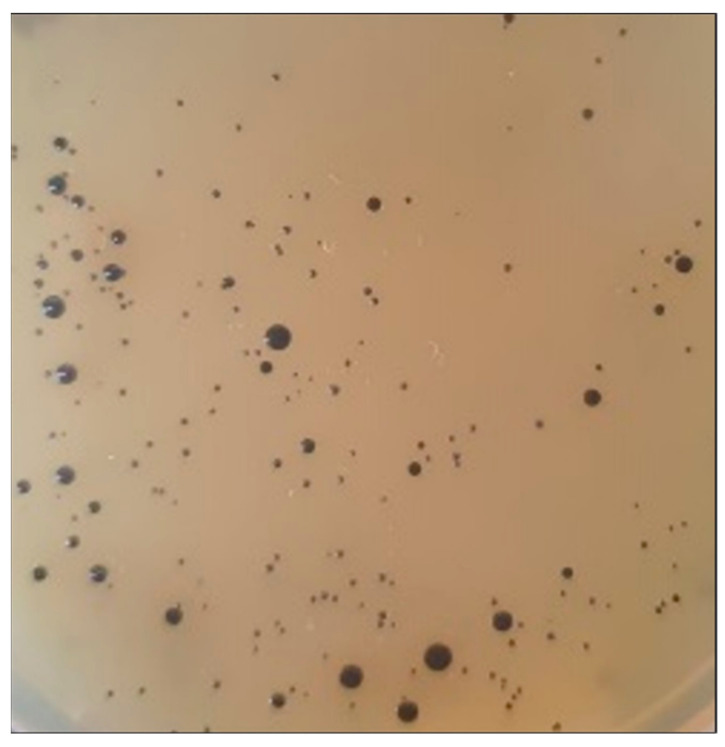
Queso de Bola 10^−2^ dilution plated in Baird Parker agar. The black colonies observed lack the characteristic halo of *S. aureus*.

Samples positive for *S. aureus* DNA were streaked on Baird Parker agar to confirm the qPCR results. No typical colonies for this pathogen were detected on any plate (Figure 6), and due to the detection limit of the culture-dependent test, the plate count was <100 CFU/g cheese, which is within the limit established for ripened cheeses in the Mexican Official Standard for dairy products [6].

*L. monocytogenes* can be found in fresh cheeses made with raw milk because it is resistant to the salt and acidity concentrations typical of this type of product [46]. This bacterium causes listeriosis, a relevant disease in susceptible populations, such as old adults, infants, pregnant women, and immunocompromised people [47,48,49]. Thermal processes in milk or curd can eliminate it, but its ability to produce biofilms allows it to persist in food processing equipment [31,48,50,51]. Our results from the 111 samples analyzed show the absence of *L. monocytogenes* DNA, indicating that this bacterium is not present in the vessels and appliances used for cheese production.

*Brucella abortus* could be present in products made with raw milk from infected cows, so maintaining herd health is essential. In Mexico, the Federal Law on Animal Health continuously encourages the participation of farmers in the Program for the Verification of Bovine Brucellosis- and Tuberculosis-Free Herds through national vaccination campaigns in order to comply with the Mexican Standard NOM-041-ZOO-1995 (National Campaign against Brucellosis in Animals) [52,53]. Furthermore, the regulations that apply to Cotija cheese establish that cattle shall be free of *Brucella* and *Mycobacterium* [7]. Brucellosis in humans is a zoonotic disease that, in the past decade, has generated outbreaks in the United States linked to the consumption of fresh cheeses made with unpasteurized milk illegally imported to the United States from Mexico [13]. The trade of Bola de Ocosingo and Cotija cheeses is relevant locally and with neighboring countries, especially in the United States, due to significant immigration. The recommendation would be to produce these cheeses using pasteurized milk or milk from healthy cattle; however, the ripening process is also relevant to avoid its presence in raw milk cheeses [54]. Our results indicate the absence of DNA from this pathogenic bacterium in both cheeses, suggesting that the dairy cattle in the producing areas are healthy. In Cotija cheese production, this reflects that cheese producers follow the Mexican Official Standard for this product, and since they are cattle owners, they ensure their herd health by participating in vaccination campaigns [52].

Gastrointestinal foodborne diseases caused by *E. coli* ETEC are among the most common in the world, and the O157:H7 type is of great concern due to the mortality it causes [55,56]. This bacterium is also common in milk and dairy products, causing outbreaks worldwide [57,58,59]. Milk pasteurization is the best procedure to eliminate coliforms; however, good manufacturing practices are paramount. During the ripening of cheeses made from unpasteurized milk, the decrease in pH, loss of moisture (decrease in water activity, a_W_), and microbiological competition processes, such as the action of bacteriocins, can reduce coliform populations [60]. Studies on the ripening of Cotija cheese showed that the coliform count decreases drastically during the first 60 days of ripening. This finding supports the view that ripening is essential to improve the microbiological quality of cheeses made with unpasteurized milk [12,61]. However, eliminating this enterobacteria during ripening is not always possible, so its absence in milk is essential [55]. Our results showed that *E. coli* O157:H7 or ETEC was absent from the 111 samples, indicating that hygiene processes were followed.

Our research group performed a metagenomic analysis of the same batch of Cotija cheese samples analyzed in this work. The massive sequencing results showed the absence of *L. monocytogenes*, *E. coli* O157:H7, ETEC, or *Brucella* spp. DNA sequences, which was consistent with the results of the qPCR assays [8]. These studies also showed the presence of the *Staphylococcus* genus as a subdominant population, which could explain the presence of atypical colonies in the Baird-Parker plate. Culture-dependent studies of lipolytic strains from Cotija cheese in our group allowed us to isolate strains of *S. xylosus*, *S. piscifermentans*, and *S. saprophyticus*, among others. [62]. On the other hand, Aldrete-Tapia et al. investigated the metagenome composition of Bola de Ocosingo cheese, and they reported the presence of low proportions of *E. coli* DNA; however, they could not recover colonies by traditional microbial analysis, indicating the presence of non-cultivable cells [63].

Another enterobacterium of concern is *Salmonella*, which can be found in milk and cheese due to poor hygiene procedures. It is one of the main causes of foodborne outbreaks worldwide, and its tolerance is zero in most countries’ regulations [64]. Notably, the presence of *S. aureus* in cheese may indicate unsanitary handling during its production, or it could come in milk from cows with mastitis. Its presence at high concentrations (≥10^5^ CFU/g) causes food poisoning by ingesting the enterotoxins it produces [38,65]. Since *S. aureus* strains from raw milk cheeses can produce enterotoxins A, B, C, D, and E, it would be interesting to detect by PCR the presence of the genes encoding them (*sea*, *seb*, *sec*, *sed*, and *see*, respectively) in samples that tested positive for *S. aureus* by qPCR [66]. Our analyses revealed the presence of DNA from these foodborne pathogens in some samples, as we observed amplification curves with Ct values between 23 and 30. These results suggest that those microorganisms may have been present at some point; however, changes in the physicochemical conditions of the cheese during the ripening process inhibited their development, and they were no longer viable according to the results shown in Figure 3, where the Ct values were similar to those obtained with the non-viable inoculum. This finding is supported by the absence of *S. aureus* in the plate count (Figure 6).

In 2021, Dr. Torres-Vitela’s research group spiked milk used to produce Cotija cheese with known concentrations of *S. aureus*, *L. monocytogenes*, and *S. enterica* Typhimurium to monitor the effect of the ripening process on the survival of these microorganisms in cheese. By plate culturing and biochemical tests, their results showed the inhibition of *L. monocytogenes* between days 15 and 30 of ripening and *S. aureus* and *S. enterica* between days 30 and 45; furthermore, no staphylococcal toxin was detected at any stage of the ripening process. Their results support the importance of the ripening process in the safety of the final product [12].

The information mentioned above supports that the ripening process in both types of cheese is relevant not only for obtaining their characteristic flavor and aroma but also plays a central role in the safety of the finished product; the development of lactic acid bacteria, together with the decrease in pH and a_W_, inhibits the proliferation of coliform bacteria, pathogens, and bacteria susceptible to stress conditions.

## 4. Conclusions

The qPCR analysis was a rapid and effective tool to detect pathogenic bacteria in ripened Cotija and Bola de Ocosingo artisanal cheeses, confirming its value in assessing the microbiological safety of these cheeses. We suggest using this method as a screening test with positive samples subsequently analyzed by culture-dependent methods. Due to the commercial and cultural importance of cheeses made with raw milk, it is relevant to emphasize that the good microbial quality of milk and other raw materials and the use of hygienic processes and sanitized facilities and tools are of the utmost importance. Our results highlight the relevance of carrying out the ripening process for a sufficient time to eliminate any pathogens that may be incorporated into the product, as its manufacture involves multiple manual processes. Ripened cheeses made with raw milk do not necessarily imply a microbiological risk; on the contrary, manufacturing products that are safe to consume and have highly acceptable organoleptic characteristics enriches the offer of dairy products. Finally, it is worth mentioning the importance of consuming cheese from farms belonging to the regions of origin. Imitation cheeses, which can be found in large-scale distribution chains, do not necessarily comply with the production and ripening rules stipulated for authentic ones, and this could lead to the consumption of products of dubious origin and innocuousness.

## Figures and Tables

**Table 1:** Culture medium and culture conditions for each pathogenic microorganism.

Microorganism	Enrichment Broth	Culture Conditions
*Salmonella* spp.	Buffered peptone water	37 °C, 24 h, 250 rpm
*S. aureus*	Nutrient broth (OXOID, Basingstoke, UK)	37 °C, 48 h, 250 rpm
*L. monocytogenes*	Fraser broth (DIFCO, Detroit, MI, USA)	37 °C, 48 h, 250 rpm
*Brucella* spp.	Brucella broth (DIFCO, Detroit, MI, USA) + amphotericin B (1 mg/mL) (Sigma-Aldrich, St. Louis, MO, USA), and vancomycin (20 mg/mL) (Sigma-Aldrich, St. Louis, MO, USA)	37 °C, 7 days, static
*E. coli* ETEC and *E. coli* O157:H7	BHI (DIFCO, Detroit, MI, USA) + casamino acids (2%) (OXOID, Basingstoke, UK)	37 °C, 24 h, static

**Table 2:** Target genes, primers, and fluorogenic probes to detect each pathogen by qPCR.

Microorganism	Target Gene	Nucleotide Sequences 5′ → 3′	Observations/Reference
*Salmonella* *enterica*	*invA* (invasion protein)	Fw-ACCGTGGTCCAGTTTATCGTTATT Rv-GGGCATACCATCCAGAGAAAATCG FAM-TCCGCGACACGTTCTG	This work. Target gene reported by Malorny et al. [19]
*Staphylococcus aureus*	*nucA* (staphylococcal thermonuclease)	Fw-CCTGAAGCAAGTGCATTTACGAAAA Rv-CGCTAAGCCACGTCCATATTTATCA FAM-CTCGACTTCAATTTTC	This work. Target gene reported by Ruiz-Pérez et al. [20]
*Listeria* *monocytogenes*	*hly* (listeriolysin)	Fw-AAGGTGCTACTTTTAACCGGGAAA Rv-CATTGTCTTTTAAGAAGTTTGTTGTATAGGCA FAM-CACCAGGAGTTCCC	This work. Target gene reported by Kim et al. [21]
*Brucella* spp.	*per* (perosamine synthetase)	Fw-GTTTAGTTTCTTTGGGAACAAGACAA Rv-GAGGATTGCGCGCTAGCA FAM-TACGACCGGTGAAGGCGGGATG	Individual synthesis of primers and probe as reported by Bounaadja et al. [22]
*Escherichia coli* ETEC	*eltBI* (thermolabile toxin subunit B)	Fw-GAGTACTTCGATAGAGGAACTCAAATGAAT Rv-TCATCATATCTGACAAAGCCGGTTT FAM-CCTCTCGCGTGATCAT	This work. Target gene reported by Nada et al. [23]
*Escherichia coli* O157:H7	*eae* (intimin)	Fw-CATTGATCAGGATTTTTCTGGTGATA Rv-CTCATGCGGAAATAGCCGTTA VIC-ATAGTCTCGCCAGTATTCGCCACCAATACC	Individual synthesis of primers and probe as reported by the European Food Safety Authority [24]

**Table 3:** Cross-reactivity of primers and probes. Cycle threshold (Ct) values were obtained using template DNA extracted from pure cultures of the different bacteria.

Bacterium (Target Gene)	*S. enterica* Typhimurium	*S. aureus*	*L. monocytogenes*	*B. abortus*	*E. coli* ETEC	*E. coli* O157:H7	*E. faecalis*	*E. faecium*	*L.* *fermentum*	*E. coli* DH5α
*Salmonella* spp. *(invA)*	13.59 ± 0.23	>39	>39	>36	>36	>35	>33	>33	>33	>32
*S. aureus (nucA)*	>32	13.95 ± 0.53	>33	>33	>31	>31	>33	>37	>37	>33
*L. monocytogenes (hly)*	>34	>30	13.83 ± 0.70	>35	NA	NA	NA	>39	>37	>38
*Brucella* spp. *(per)*	NA	NA	NA	12.20 ± 0.09	NA	NA	NA	NA	NA	NA
*E. coli* ETEC *(eltBI)*	>34	>30	>30	>36	11.57 ± 0.29	>30	>33	>35	>37	NA
*E. coli* O157:H7 *(eae)*	>34	>31	>30	>36	>32	11.72 ± 0.69	>31	>34	>37	NA

NA—no amplification. The mean and standard deviation of four replicates are shown.

**Table 4:** Ct values with template DNA from the pure culture of the pathogen versus DNA from the bacterium inoculated in cheese.

Microorganism	Ct (DNA from Pure Bacterium Culture)	Ct (DNA from the Bacterium Spiked in Cheese)
*S. enterica*	13.59 ± 0.23	13.55 ± 0.24
*S. aureus*	13.95 ± 0.53	14.53 ± 0.02
*L. monocytogenes*	13.83 ± 0.70	13.99 ± 0.84
*B. abortus*	12.20 ± 0.09	13.17 ± 0.71
*E. coli* ETEC	11.57 ± 0.29	11.35 ± 0.65
*E. coli* O157:H7	11.72 ± 0.69	13.08 ± 1.15

The mean and standard deviation of two replicates are shown.

**Table 5:** Occurrence of DNA from foodborne bacteria detected by qPCR in cheese samples.

Microorganism	Number of Positive Samples/Total (%)	
	Cotija Cheese	Bola de Ocosingo Cheese
*S*. *enterica*	10/95 (10.5%)	0/16
*S. aureus*	13/95 (13.7%)	0/16
*L. monocytogenes*	0/95	0/16
*Brucella* spp.	0/95	0/16
*E. coli* ETEC	0/95	0/16
*E. coli* O157:H7	0/95	0/16

**Table 6:** Ct values in positive qPCR analyses of Cotija cheese.

Sample Identifier	*Salmonella* spp. Ct Value	*S. aureus* Ct Value
QC-10	>30	28.92 ± 0.77
QC-11	24.76 ± 0.85	>30
QC-12	23.54 ± 0.14	>30
QC-13	25.23 ± 0.90	>30
QC-14	23.85 ± 0.21	>30
QC-15	26.03 ± 0.52	28.15 ± 0.21
QC-16	24.75 ± 0.37	>30
QC-17	23.78 ± 0.15	>30
QC-18	24.17 ± 0.49	>30
QC-19	27.93 ± 0.63	>30
QC-20	26.17 ± 0.63	>30
QC-53	>30	29.31 ± 2.39
QC-56	>30	26.77 ± 0.21
QC-59	>30	28.61 ± 0.20
QC-61	>30	29.34 ± 1.83
QC-70	>30	29.45 ± 0.16
QC-72	>30	27.46 ± 1.98
QC-76	>30	28.94 ± 0.80
QC-77	>30	29.17 ± 0.71
QC-82	>36	29.43 ± 0.48
QC-85	>36	28.62 ± 1.13
QC-93	>30	29.20 ± 0.80

The mean and standard deviation of four replicates are shown.

## Data Availability

The original contributions presented in the study are included in the article; further inquiries can be directed to the corresponding author.
